# A Systematic Review of Therapeutic Process Factors in Mental Health Treatment for Autistic Youth

**DOI:** 10.1007/s10567-022-00409-0

**Published:** 2022-08-24

**Authors:** Carly S. Albaum, Nisha Vashi, Yvonne Bohr, Jonathan A. Weiss

**Affiliations:** Department of Psychology, York University, 4700 Keele Street, Toronto, ON M3J 1P3 Canada

**Keywords:** Systematic review, Process factors, Autism, Youth, Mental health, Psychotherapy

## Abstract

Understanding the role of therapeutic process factors in treatment change may prove useful for discerning why some autistic youth benefit from psychosocial interventions that target emotional and psychological aspects of mental health, while others do not. The aim of the current study was to synthesize what is currently known about therapeutic process factors in mental health treatment of emotional and psychological challenges for autistic youth, regarding how process factors have been measured in past research, and the relation between process factors and treatment outcome. A systematic review of the literature was conducted to narratively synthesize all articles published up until June 2021. Methodological quality of included studies was appraised. Twenty-five studies met inclusion criteria. Process factors assessed across studies included relational factors; treatment expectations, readiness, and satisfaction; and treatment engagement from youth and their parents. Process-outcome associations were reported for a limited number of constructs. There is a limited, albeit growing, body of high-quality research evaluating the role of process factors in the treatment of mental health issues for autistic youth. Future research should continue to examine process factors in relation to treatment outcome, and validate measures to accurately capture process-related constructs in mental health treatment for this population. Greater understanding of therapy processes can lead to developing evidence-informed strategies that clinicians can implement to promote positive expectations, relationships, and engagement.

**Supplementary Information:**

The online version contains supplementary material available at 10.1007/s10567-022-00409-0.

## Background

Autistic children and adolescents often experience emotional and psychological challenges related to mental health, such as anxiety, mood, and associated behavioural problems (Lai et al., 2019; Salazar et al., 2015). These mental health issues may be attributed in part to a limited capacity to regulate emotions (Mazefsky & White, 2014; Weiss, 2014) and can manifest as tantrums or meltdowns, self-injurious behaviour, or aggression towards others. In addition to the occurrence of sub-clinical mental health challenges, approximately 70% of autistic youth^1^ are estimated to meet criteria for at least one psychiatric disorder (Mattila et al., 2010; Simonoff et al., 2008), with even higher rates reported for youth receiving mental health services (Brookman-Frazee et al., 2018).

There is emerging evidence supporting the use of psychosocial interventions to address mental health challenges for verbally able autistic youth. Cognitive behaviour therapy (CBT) is the most well-researched intervention to date and has largely focused on reducing anxiety-related symptoms (Ameis et al., 2018; Weston et al., 2016). Recent research suggests CBT may also be useful in promoting emotion regulation skills (Weiss et al., 2018). Early-stage research on other psychosocial treatments, such as mindfulness-based interventions, also show promise for promoting psychological well-being in young autistic people (Hartley et al., 2019). Nonetheless, a notable portion of autistic children who take part in psychosocial interventions do not exhibit clinically meaningful improvements upon therapy completion (e.g., Wood et al., 2009, 2020). For example, a systematic review of anxiety treatment for autistic youth indicated that up to 71% of youth responded to CBT; in other words, at minimum, almost one-third of those who participated in therapy *did not* demonstrate clinically significant improvement (Vasa et al., 2014). Additionally, meta-analytic results indicate that only 23% of autistic youth who participate in CBT show complete recovery from anxiety symptoms (Warwick et al., 2017). Beyond anxiety, it has been shown that approximately half of autistic youth receiving modified CBT for obsessive–compulsive symptoms do not show meaningful improvement at the end of treatment (Jassi et al., 2021), and one-third have been deemed non-responders in regard to changes in emotion regulation (Swain et al., 2019). Understanding the specific factors that contribute to treatment success may prove useful for discerning *who* benefits from treatment and *why*, and could potentially lead to enhanced treatment effectiveness for youth who are particularly susceptible to mental health challenges.

Common therapeutic factors may play a role in some of the variation observed in mental health outcomes for autistic youth who take part in psychosocial interventions. The common factor approach recognizes the importance of therapy-specific techniques for effecting change (e.g., thought records or behavioural activation in CBT), but also emphasizes the influence of factors that are common across therapeutic modalities (Thomas, 2006). For example, client pre-treatment characteristics, such as symptom severity, expectations about therapy, or developmental level, are believed to impact the therapeutic process, regardless of the type of therapy (e.g., CBT, psychodynamic; Karver et al., 2005). *Process factors* are a subgroup of common factors that broadly refer to universal aspects of treatment, which unfold from moment to moment over the course of therapy (Orlinsky, 2001). At the individual level, these factors may include client behaviour within treatment sessions, such as the quality of participation in-session tasks, and outside the therapy environment, such as adherence to at-home skill practice. The therapeutic process may be influenced by pre-treatment factors that evolve and become part of the therapy process, such as treatment expectation or willingness to participate. Interpersonal factors, such as the working relationship between the client and therapist, known as the therapeutic alliance, or parental scaffolding during home practice (in the case of child-focused therapies) may also affect the therapy process. Process factors are believed to contribute to symptom change, beyond unique technical elements of any specific therapeutic modality (Brown, 2015; Sprenkle & Blow, 2004).

There is a growing interest in process-related factors within the field of youth-focused therapy. Over the past two decades, several reviews and meta-analyses have been conducted to summarize process-related constructs in association with youth treatment outcome (Becker et al., 2018; Fjermestad et al., 2009; Karver et al., 2006, 2018; Kazantzis et al., 2016). Process factors that have been assessed in the youth treatment literature include therapeutic alliance with both youth and parent, youth and parent willingness to participate in treatment, youth and parent involvement, as well as therapist-specific factors, such as use of self-disclosure, therapist experience, and perceived competency. Relationship factors are the most well-understood process factor in the youth literature outside of autism-related research. The latest meta-analytic review (Karver et al., 2018) identified 28 studies examining the association between therapeutic alliance and treatment outcome, which yielded an overall moderate-sized effect—in line with previous alliance-outcome associations observed in youth-focused therapies (Karver et al., 2006; Shirk & Karver, 2011). Several studies have also examined youth treatment expectations and motivation to participate in therapy, which have been shown to predict whether youth complete treatment or terminate early, and which are positively related to symptom reduction in mental health-related outcomes (Adelman et al., 1984; Dew-Reeves & Athay, 2012; Lewin et al., 2011; Wergeland et al., 2015). Treatment engagement (interchangeably referred to as “involvement” or “participation”) encompasses participation both within and outside of therapy sessions. Based on findings from 13 studies, meta-analysis of in-session participation and treatment adherence (i.e., homework completion) in the youth treatment literature indicated an overall moderate-sized association between child participation and treatment outcome (Karver et al., 2006).

In addition to youth-focused process factors, numerous studies have examined parent-focused factors that may contribute to therapeutic change. Parents’ willingness to participate in their children’s treatment has been found to be a moderate-sized predictor of youth improvement (Karver et al., 2006). Therapeutic alliance between parents and therapist, and parent participation in and out of sessions, predicts a small portion of variance in youth mental health outcomes (Karver et al., 2006; McLeod, 2011). Across youth and parent-related process factors, small to moderate-sized effects have been reported for process-outcome associations within the youth treatment literature (Karver et al., 2006, 2018).

Findings from existing studies largely apply to youth treatment in general and have often failed to specify whether any of the included research involved autistic youth. It is unclear whether similar effect sizes are observed, or whether process factors can be validly and reliably measured in the context of therapy for this population. It is plausible that the therapeutic process unfolds differently for autistic youth, relative to peers without autism, because of common difficulties with social-communication and restrictive patterns of thinking, as well frequent co-occurring challenges, like inattention, executive dysfunction, and oppositionality (Demetriou et al., 2018; Salazar et al., 2015). For example, therapeutic alliance may have a disparate relation to treatment outcome for youth who struggle with social relationships. Given how commonly occurring mental health problems are (Lai et al., 2019) and the marked portion of autistic youth who do not improve from psychotherapy (e.g., Vasa et al., 2014), it is important to establish a strong knowledge base on addressable factors that can contribute to therapeutic success for this population. The aim of the current study was to synthesize what is currently known about therapeutic process factors in mental health treatment for autistic youth, in regard to how process factors have been measured in past research, and the relation between process factors and treatment outcome.

## Method

### Search Strategy

A systematic review of empirical research examining process factors in mental health treatment for autistic youth was conducted in accordance with the standards described by the Preferred Reporting Items for Systematic Reviews and Meta-Analysis (PRISMA; Moher et al., 2009) guidelines. The review protocol was prospectively registered with the PROSPERO international review database (ID: CRD42021240272; https://www.crd.york.ac.uk/prospero/). A concurrent search of Ovid MEDLINE, PubMed, and PsycINFO was conducted on June 7, 2021 to identify all articles published to date. The final search strategy is provided in Supplemental Table 1. Based on the previous review of therapeutic process factors completed by Karver et al. (2006), 29 process-related search terms were selected. The search strategy also included autism-related terms (i.e., autis*, Asperger syndrome) and terms describing youth age range (i.e., child*, pediatric, youth, adolescen*, and kid), which were combined with the process-related terms. When possible, terms were modified to align with database-specific index terms (e.g., MeSH terms for PubMed).

### Selection Criteria

Studies identified through the searches were included based on the following criteria: (a) evaluated or described a psychosocial intervention addressing an emotional or psychological mental health-related outcome (as either a primary or secondary outcome; e.g., anxiety, depression); (b) at least a portion of the sample included youth under the age of 18 years, with a previous diagnosis of autism or related disorder (e.g., Asperger syndrome); (c) assessed and/or described at least one process-related factor; and (d) was available in English. Exclusion criteria were: (a) interventions addressing disruptive behaviour only (e.g., opposition, defiance, conduct problems) without consideration of the emotional and psychological aspects of mental health; (b) non-empirical publications (e.g., editorials, commentaries, books, book chapters, theoretical papers); (c) reviews or meta-analyses; and (d) unpublished dissertations or theses. Notably, there is a large body of literature (e.g., studies evaluating applied behavioural interventions) that has focused on reducing “disruptive” behaviours (e.g., self-injurious behaviour; aggression towards others) without evaluating the role of internalized emotional and psychological experiences (e.g., anxiety, depression, emotion regulation) that may explain the observable behaviour. Although it is not uncommon for autistic youth to demonstrate disruptive behaviour that stems from emotional and psychological issues, it is uncertain whether these problems are a targeted treatment outcome of an intervention when there is a lack of direct measurement. For clarity and consistency, the decision was made to focus on studies that explicitly assessed emotional and psychological outcomes related to mental health.

To identify eligible studies, all titles and abstracts of articles retrieved through database searches were independently screened by two authors (CA and NV). For the screening phase, disagreements were resolved through discussion until consensus was reached between the two authors. All articles that underwent full-text review were also independently reviewed by each author. Disagreements for full-text review were resolved through discussion and consultation with the senior author (JW).

### Quality Appraisal of Included Studies

The Mixed Methods Analysis Tool (MMAT; Hong et al., 2018) was used to assess the methodological quality of studies that met inclusion criteria, which was developed for empirical studies based on the study design. To determine whether a study is considered empirical, reviewers respond “Yes” or “No” to two screening questions regarding clarity of research questions and adequacy of data collection for addressing research questions. For studies that fail to meet screening criteria, quality appraisal is not conducted. For articles that meet screening criteria, reviewers respond “Yes” or “No” to five methodological-related questions that vary depending on study design (e.g., qualitative vs. randomized controlled trial). If there is insufficient information provided in the article to answer the question, reviewers may respond with “Can’t Tell”. As per the MMAT guidelines (Hong et al., 2018), methodological quality of each study is considered based on responses to individual criterion, versus a global rating or overall score. Included studies were assessed by one of two reviewers (CA or NV), and approximately 30% of studies (*n* = 8) were coded by the other reviewer as a reliability check. Reviewers were recused from rating articles that they authored. There was substantial agreement across raters (Fleiss κ = 0.77). Disagreements in ratings were resolved through discussion. The MMAT has been used as a quality appraisal tool in other review studies focused on developmental disabilities literature, including autism (Albaum et al., 2021; Doherty et al., 2020).

## Results

### Search Results

**Fig. 1:**
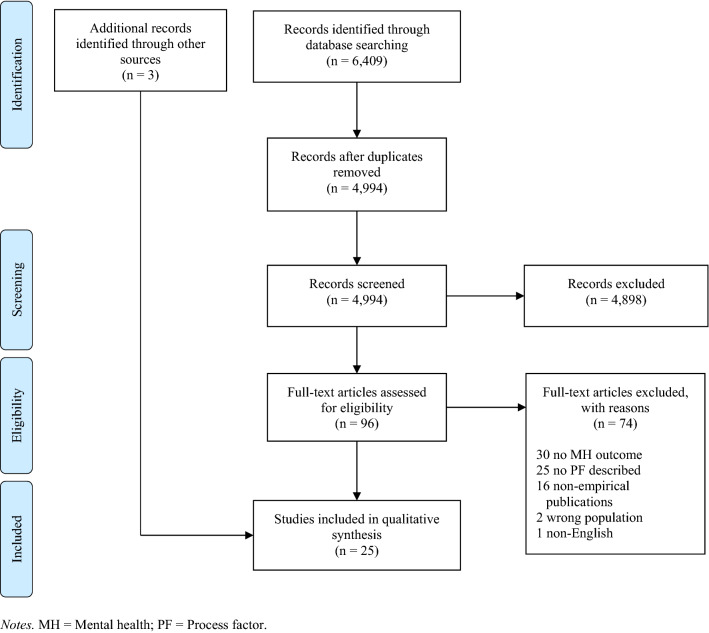
PRIMSA flow diagram illustrating study selection

Search results are illustrated in Fig. 1. An initial search of the databases using the search strategy described above yielded 6,409 articles. After duplicate articles were removed, 4,994 articles remained for review. The two authors screening titles and abstracts agreed on whether studies should be excluded or undergo full-text review for 97% of articles (*n* = 4849 articles). Title and abstract screening resulted in 4,898 articles being excluded. The full-text for the remaining 96 articles were again reviewed by both authors. Authors agreed regarding eligibility for 82% of articles (*n* = 79). Full-text review resulted in an additional 74 articles being excluded (see Fig. 1 for exclusion reasons), leaving 22 articles for data extraction and synthesis. Reference lists from included articles were reviewed for potentially relevant studies that were not identified in the original search, which resulted in inclusion of three additional studies (Brown et al., 2015; Jassi et al., 2021; White et al., 2013).

### Study Characteristics

Study characteristics are detailed in Table 1, including study location, study design, sample characteristics, description of intervention, mental health outcomes, and process factors identified. Twenty-five studies derived from 21 unique samples were identified; four of the included articles utilized the same sample (Albaum et al., 2020; Burnham Riosa et al., 2019; Thomson et al., 2015; Weiss et al., 2018). Although there was no lower date bound set for inclusion criteria, all studies identified through the search were published from 2012 onwards, with 68% of studies (*n* = 17) being published within the past six years (i.e., after 2015). Of the 25 included studies, 64% (*n* = 16) were conducted in North America and 28% (*n* = 7) in Europe; one study occurred in Asia, and one study occurred in Australia. In terms of study design, most studies were quantitative-descriptive in nature (36%, *n* = 9). Five studies (20%) conducted quantitative, non-randomized intervention trials, and five studies were randomized controlled trials. Three studies (12%) employed qualitative designs, and the remaining three studies used a mixed methods approach, involving quantitative and qualitative methods.

**Table 1:** Characteristics of included studies

Study	Country	Study design	Intervention description	Sample description	Process factor description	
					PF described/assessed	PF examined in relation to outcome?
Albaum et al. (2020)	Canada	Quantitative, descriptive	Intervention: CBT Dosage: 10 weekly sessions, 60 min. each (first session, 90 min.) Format: Individual; Full parent involvement Setting: University Provider: Three post-doctoral fellows and 19 graduate students MH Outcome: Emotion regulation	*n*: 48 Age: 8 to 12 years (*M* = 9.60, *SD* = 1.25) Gender: 92% male Dx confirmation: Diagnostic report provided; SCQ and SRS-2; ADOS administered when report unavailable (*n* = 2)	Treatment readiness; Youth-therapist alliance	Yes
Backman et al. (2018)	Sweden	Mixed methods	Intervention: Psychoeducation Dosage: 8 modules Format: Internet-based; Weekly access to modules; Weekly check-ins with clinician; No parent involvement Setting: Virtual Provider: Clinicians ("coaches") MH Outcome: Anxiety, depression	*n*: 28 Age: 16 to 25 years (*M* = 20.62; *SD* = 2.60) Gender: 57% female Dx confirmation: Diagnostic report provided; Dx verified by clinical psychologist or OSU Autism Rating Scale	Treatment expectations; Treatment satisfaction	No
Brewe et al. (2021)	USA	Quantitative, descriptive	Intervention: Mindfulness-based intervention Dosage: 16 weekly sessions, 45 to 60 min. each Format: Individual; No parent involvement Setting: University, multi-site Provider: Three clinical psychologists, one post-doctoral fellow, two master's-level clinicians, nine graduate students MH Outcome: Anxiety, depression, emotion regulation	*n*: 37 Age: 12 to 21 years (*M* = 15.28; *SD* = 2.21) Gender: 78% male Dx confirmation: ADOS	Youth-therapist alliance	Yes
Brown et al. (2015)	UK	Quantitative, descriptive	Intervention: CBT or counselling Dosage: NR Format: Individual; Partial parent involvement Setting: Child and adolescent mental health centres, multi-site Provider: Three consultant child psychiatrists, one clinical psychologist, and one counsellor MH Outcome: Anxiety	*n*: 13 Age: 12 to 18 years (*M* = 15.23; *SD* = 1.24) Gender: 54% male Dx confirmation: ADOS and ADI-R	Youth-therapist alliance	No
Burnham Riosa et al. (2019)	Canada	Quantitative, descriptive	Intervention: CBT Dosage: 10 weekly sessions, 60 min. each (first session, 90 min.) Format: Individual; Primary caregiver involved for full duration of all sessions Setting: University Provider: Graduate students and post-doctoral fellows MH Outcome: Emotion regulation	*n*: 20 Age: 8 to 12 years (*M* = 9.75; *SD* = 1.29) Gender: 95% male Dx confirmation: Diagnostic report provided; SCQ and SRS-2	Parent-therapist alliance; Treatment adherence; Youth involvement; Youth-therapist alliance	No
Chlebowski et al. (2018)	USA	Qualitative	Intervention: Psychotherapy/counselling Dosage: NR Format: Individual; Parent-mediated Setting: Community-based mental health Provider: 17 therapists (59% staff, 41% trainees; 18% licensed in clinical discipline) MH Outcome: Challenging behaviour, psychiatric comorbidities	*n*: 29 Age: Age range NR (*M* = 9.8 years; *SD* = 2.06) Gender: 90% male Dx confirmation: NR	Parent involvement; Parent-therapist relationship	NA
Drmic et al. (2017)	Singapore	Mixed methods	Intervention: CBT Dosage: 10 weekly sessions, 60 to 90 min. each Format: Small groups (2–3 youth); Partial parent involvement Setting: Mainstream secondary school Provider: 23 allied educators trained to facilitate intervention; 19 supervising psychologists ("coaches") MH Outcome: Anxiety	*n*: 44 Age: 13 to 15 years (*M* and *SD* NR) Gender: 86% male Dx confirmation: “Known diagnosis” made using clinical practice guidelines (i.e., use of ADOS/ADI-R)	Parent involvement; Therapist direct influence skills Treatment readiness; Youth motivation	NA
Edgington et al. (2016)	UK	Mixed methods	Intervention: CBT Dosage: 8 weekly sessions, 45 min. each Format: Group; Email communication with parent-only Setting: Mainstream secondary school Provider: NR MH Outcome: Anxiety	*n*: 7 Age: 11 to 16 years (*M* = 13.91; *SD* = 1.45) Gender: 100% male Dx confirmation: Statement of Special Education Needs (SEN) provided for "most" participants	Group cohesion; Parent involvement; Youth motivation	NA (process factors result of qualitative analysis)
Gordon et al. (2015)	UK	RCT	Intervention: Psychoeducation Dosage: 6 weekly session, 90 min. each Format: Group; Parallel parent-only sessions Setting: NR Provider: Clinical psychologists MH Outcome: Psychopathology, self-esteem	*n*: 48 Age: 9 to 14 years (*M* = 11.45; *SD* = 1.55) Gender: 83% male Dx confirmation: 3Di-sv	Treatment adherence; Treatment satisfaction	No
Hillier et al. (2012)	USA	Non-randomized	Intervention: Music program Dosage: 8 weekly sessions, 90 min. each Format: Group; No parent involvement Setting: University Provider: Music education and psychology students (with professor supervision) MH Outcome: Anxiety, self-esteem	*n*: 22 Age: 13 to 29 years (*M* = 18, *SD* NR) Gender: 82% male Dx confirmation: Proof of prior diagnosis required for eligibility	Treatment satisfaction	No
Jassi et al. (2021)	UK	Non-randomized	Intervention: CBT with ERP Dosage: 14 to 30 sessions (Mode = 20); Frequency and duration NR Format: Individual; Partial parent involvement Setting: Mental health clinic + trigger-related environments (e.g., home) Provider: Clinical psychologists MH Outcome: OCD symptoms	*n*: 34 Age: 11 to 17 years (*M* = 15.18; *SD* = 1.70) Gender: 68% male Dx confirmation: ADOS and/or ADI-R for 68% of sample	Family accommodation; Treatment satisfaction	No
Jones and Jassi (2020)	UK	Qualitative	Intervention: CBT Dosage: 20 sessions over 24 weeks, 60 min. each Format: Individual; Full parent involvement + 6 parallel parent-only sessions Setting: Clinic- and home-based Provider: Two clinical psychologists MH Outcome: OCD symptoms	*n*: 1 Age: 16 Gender: Male Dx confirmation: NR	Family accommodation	No
Kang et al. (2021)	USA	Quantitative, descriptive	Intervention: Social skills intervention Dosage: 5 h. per day, 5 days per week for 6 weeks Format: Group; No parent involvement Setting: Community-based Provider: Head therapist and two support therapists; Supervised by MA-level therapist MH Outcome: Social anxiety	*n*: 34 Age: 9 to 16 years (*M* = 12.41*, SD* = 2.06) Gender: 79% male Dx confirmation: SCQ or SRS-2	Youth-therapist alliance	Yes
Kerns et al. (2018)	USA	Quantitative, descriptive	Intervention: CBT Dosage: 16 weekly sessions, 60 to 90 min. each Format: Individual; Partial parent involvement Setting: University Provider: NR MH Outcome: Anxiety, internalizing and externalizing problems	*n*: 64 Age: 7 to 16 years (*M* = 10.81, *SD* = 2.25) Gender: 81% male Dx confirmation:—ADOS, ADI-R, and clinical judgment	Parent-therapist alliance; Youth-therapist alliance	Yes
Klebanoff et al. (2019)	USA	Quantitative, descriptive	Intervention: CBT Dosage: 16 to 32 sessions (time duration NR), 90 min. each Format: Individual; Full parent involvement Setting: NR Provider: Two doctoral-level psychologists; 11 graduate students MH Outcome: Anxiety	*n*: 64 Age: 5 to 15 years (*M* = 10; *SD* = 2.0) Gender: 77% male Dx confirmation: ADOS and ADI-R	Parent-therapist alliance; Youth-therapist alliance	Yes
London et al. (2020)	Australia	Qualitative	Intervention: Animal-assisted occupational therapy involving dogs Dosage: Five weekly sessions, 60 min. each Format: Individual; Full parent involvement Setting: Assistance Dogs Australia Provider: Occupational therapists; Four assistance dog trainers MH Outcome: Emotion regulation	*n*: 17 Age: 4 to 19 years (*M* = 8.88; *SD* = 4.32) Gender: 94% male Dx confirmation: NR	Youth engagement	NA
Lordo et al. (2017)	USA	Non-randomized	Intervention: Social skills intervention Dosage: 14 weekly sessions, 90 min. each Format: Group; Parallel parent-only sessions Setting: NR Provider: NR MH Outcome: Emotion regulation, internalizing and externalizing symptoms, positive and negative affect	*n*: 16 Age: 12 to 17 years (*M* = 15.07; *SD* = 1.40) Gender: 75% male Dx confirmation: GARS-3	Treatment adherence	No
McNally Keehn et al. (2013)	USA	RCT	Intervention: CBT Dosage: 16 weekly sessions, 60 to 90 min. each Format: Individual; Two parent-only sessions Setting: University Provider: Clinical psychologist MH Outcome: Anxiety	*n*: 22 Age: 8 to 14 years (*M* = 11.26; *SD* = 1.53) Gender: 95% male Dx confirmation: ADOS and ADI-R	Treatment adherence	No
Pahnke et al. (2014)	Sweden	RCT (quasi-experimental)	Intervention: ACT Dosage: 12 biweekly sessions (i.e., over six weeks), 40 min. each; 6–12 min. mindfulness exercise daily Format: Group; No parent involvement Setting: Specialized secondary schools, multi-site Provider: Graduate student supervised by ACT therapist; Classroom teachers MH Outcome: Emotional and behaviour problems, psychological distress, stress-related behaviour	*n*: 28 Age: 13 to 21 years (*M* = 16.5; *SD* = 2.0) Gender: 75% male Dx confirmation: NR	Treatment adherence; Treatment satisfaction	No
Storch et al. (2015)	USA	Quantitative, descriptive	Intervention: CBT Dosage: 16 weekly sessions, up to 90 min. each Format: Individual; Full parent involvement Setting: NR Provider: Clinical psychologists, post-doctoral fellows, graduate students MH Outcome: Anxiety	*n*: 24 Age: Age range NR (*M* = 10.42; *SD* = 2.55) Gender: 79% male Dx confirmation: ADOS and ADI-R; Review of records	Family accommodation	Yes
Swain et al. (2019)	USA	Non-randomized	Intervention: CBT Dosage: Nine weekly sessions, 60 min. each Format: Group; Parallel parent sessions Setting: University-associated community clinic; Hospital Provider: Masters and doctoral-level clinicians; Supervised by clinical psychologist MH Outcome: Emotional problems, emotion regulation	*n*: 18 Age: 4 to 7 years (*M* = 6.16; *SD* = 0.99) Gender: 89% male Dx confirmation: NR	Treatment satisfaction	Yes
Thomson et al. (2015)	Canada	Non-randomized	Intervention: CBT Dosage: 10 weekly sessions, 60 min. each (first session, 90 min.) Format: Individual; Full parent involvement Setting: University Provider: Post-doctoral fellow and four graduate students MH Outcome: Anxiety, emotion regulation, internalizing and externalizing problems	*n*: 14 Age: 8 to 12 years (*M* = 10.40; *SD* = 1.30) Gender: 93% male Dx confirmation: Diagnostic report provided; SCQ and SRS-2; ADOS administered when report unavailable	Parent-therapist alliance; Treatment adherence; Treatment satisfaction; Youth involvement; Youth-therapist alliance	No
Walsh et al. (2018)	USA	Quantitative, descriptive	Intervention: CBT Dosage: 14 weekly sessions, 90 min. each Format: Multifamily groups; Full parent involvement Setting: University-affiliated outpatient clinics, multi-site Provider: 34 mental health professionals (e.g., clinical/counselling psychologists; social workers) and graduate students MH Outcome: Anxiety	*n*: 80 Age: 8 to 14 years (*M* = 11.11; *SD* = 1.97) Gender: 84% male Dx confirmation: ADOS and SCQ	Treatment satisfaction	Yes
Weiss et al. (2018)	Canada	RCT	Intervention: CBT Dosage: 10 weekly sessions, 60 min. each (first session, 90 min.) Format: Individual; Full parent involvement Setting: University Provider: Post-doctoral fellows and graduate students MH Outcome: Anxiety, emotion regulation, internalizing and externalizing problems	*n*: 68 Age: 8 to 12 years (*M* = 9.75; *SD* = 1.27) Gender: 88% male Dx confirmation: Diagnostic report provided; SCQ and SRS-2; ADOS administered when report unavailable	Treatment adherence; Treatment satisfaction; Youth involvement	No
White et al. (2013)	USA	RCT	Intervention: CBT Dosage: 12–13 individual sessions, 60–70 min. each; 7 group sessions, 75 min. each (frequencies NR) Format: Individual + group; Partial parent involvement Setting: University-affiliated clinic Provider: Clinical psychologist and four graduate students MH Outcome: Anxiety	*n*: 30 Age: 12 to 17 years (*M* = 15; *SD* NR) Gender: 77% male Dx confirmation: ADOS and ADI-R	Treatment adherence; Treatment satisfaction; Youth involvement	No

*3Di-sv* Developmental Diagnostic Dimensional Interview, short version, *ACT* Acceptance and commitment therapy, *ADI-R* Autism Diagnostic Interview, Revised, *ADOS* Autism Diagnostic Observation Schedule, *CBT* cognitive behaviour therapy, *Dx* autism diagnosis, *ERP* exposure and response prevention, *GARS-3* Gilliam Autism Rating Scale, Third Edition, *MH* mental health, *NA* not applicable, *NR* not reported, *OCD* obsessive compulsive disorder, *PF* process factor, *RCT* randomized controlled trial, *SCQ* social-

Communication Questionnaire, *SRS-2* Social Responsiveness Scale, Second Edition

Across studies, participants ranged in age from 4 to 29 years. Nine studies (36%) involved adolescents (i.e., 13 years of age or older) and nine included both children and adolescents; five studies (20%) included children only (i.e., 12 years of age or younger), and two did not report on the age range of the sample. The mean age reported across studies was 12.6 years (*SD* = 3.41; range: 6.2 to 20.6). On average, samples comprised 82.3% males (*SD* = 13.2%, range: 43% to 100%). Sixteen studies (64%) reported that the majority of participants in the sample identified as White/Caucasian, 11 of which included samples with more than 75% White/Caucasian participants; one study sample comprised 100% Asian participants (i.e., Chinese, Indian, or Malays; Drmic et al., 2017), and one study comprised 100% Latinx participants (Chlebowski et al., 2018). Seven studies (28%) did not report on the ethnicity or racial identity of participants. Most studies (80%, *n* = 20) reported using autism diagnostic (e.g., ADOS; ADI-R) and/or screening tools (e.g., SCQ; SRS-2), or having participants share a report from a licensed healthcare provider to confirm autism diagnostic criteria were met. The majority of studies excluded participants with a diagnosed intellectual disability or who had limited intellectual abilities (e.g., IQ < 70; 76%, *n* = 19); five studies (20%) did not specify level of intellectual functioning. One study included a single participant with an intellectual disability, as reported by parents (London et al., 2020).

Regarding the interventions, the majority of studies (64%, *n* = 16) involved treatment programs that were based on CBT. Other forms of psychosocial treatment included psychoeducation (Backman et al., 2018; Gordon et al., 2015), social skills interventions (Kang et al., 2021; Lordo et al., 2017; White et al., 2013), mindfulness-based interventions (Brewe et al., 2021; Pahnke et al., 2014), counselling or psychotherapy (Brown et al., 2015; Chlebowski et al., 2018), music programs (Hillier et al., 2012), and animal-assisted occupational therapy (London et al., 2020). Fifteen studies (60%) involved individualized treatment and 36% (*n* = 9) used group formats; one study involved a combination of individual and group sessions (White et al., 2013). Parents were fully involved in treatment (i.e., were present for the entire duration of all sessions) in 40% of studies (*n* = 10), and partially involved for another 40% of studies (e.g., present for only a portion of each session; attended some, but not all sessions; participated in parallel parent-only sessions). For 20% of studies (*n* = 5), the extent of parent involvement was not reported. Mental health outcomes that were commonly targeted included anxiety (60% of studies, *n* = 15), emotion regulation (28%, *n* = 7), internalizing and externalizing symptoms broadly (16%, *n* = 4), depression (8%, *n* = 2), and obsessive–compulsive symptoms (8%, *n* = 2). Studies also examined other mental health-related outcomes such as general psychopathology and psychiatric comorbidity, psychological distress and stress-related behaviour, emotional and behaviour problems, positive and negative affect, and self-esteem.

### Quality of Included Studies

Study quality was appraised using the MMAT (Hong et al., 2018). All studies were deemed to have clear research questions and collected appropriate data to address the research questions, and thus met MMAT screening criteria to proceed for further appraisal. Eight studies (32%) met 100% of the MMAT criteria for the respective study design; 10 studies (40%) met 80% of criteria; four studies (16%) met 60% of criteria; one study (4%) met 40% of criteria; and two studies (8%) met 20% of criteria. Summary of responses to questions regarding methodological quality are provided in Table 2.

**Table 2:** Quality appraisal of included studies based on Mixed Methods Appraisal Tool (Hong et al., 2018)

Study (by design)	Methodological quality criteria				
*Qualitative studies*	Qualitative approach appropriate to answer research question?	Data collection methods adequate to answer research question?	Findings adequately derived from data?	Interpretation of results sufficiently substantiated by data?	Coherence between qualitative data, sources, collection, analysis, and interpretation?
Chlebowski et al. (2018)	Y	Y	Y	Y	Y
Jones and Jassi (2020)	Y	Y	Y	Y	Y
London et al. (2020)	Y	Y	Y	Y	Y

Randomized controlled trials	Randomization appropriately performed?	Groups comparable at baseline?	Complete outcome data?	Outcome assessors blinded to the intervention provided?	Participants adhered to assigned intervention?
Gordon et al. (2015)	Y	Y	Y	N	Y
McNally Keehn et al. (2013)	Y	Y	Y	N	Y
Pahnke et al. (2014)	Y	Y	Y	N	Y
Weiss et al. (2018)	Y	Y	Y	N	Y
White et al. (2013)	Y	Y	Y	N	Y

Non-randomized studies	Participants representative of target population?	Measurements appropriate for both outcome and intervention?	Complete outcome data?	Confounders accounted for in design/analysis?	Intervention administered as intended?
Hillier et al. (2012)	Y	Y	N	Y	Y
Jassi et al. (2021)	Y	Y	Y	Y	Y
Lordo et al. (2017)	Y	Y	Y	N	Y
Swain et al. (2019)	Y	Y	Y	Y	Y
Thomson et al. (2015)	Y	Y	Y	Y	Y

Quantitative descriptive studies	Sampling strategy relevant to research question?	Sample representative of target population?	Measurements appropriate?	Risk of nonresponse bias low?	Statistical analysis appropriate to answer research question?
Albaum et al. (2020)	Y	Y	Y	N	Y
Brewe et al. (2021)	Y	Y	Y	Y	Y
Brown et al. (2015)	Y	Y	Y	N	Y
Burnham Riosa et al. (2019)	C	Y	Y	N	Y
Kang et al. (2021)	Y	C	Y	N	Y
Kerns et al. (2018)	Y	Y	Y	N	Y
Klebanoff et al. (2019)	N	Y	Y	N	Y
Storch et al. (2015)	N	Y	Y	N	Y
Walsh et al. (2018)	Y	Y	Y	Y	Y

Mixed methods studies	Adequate rationale for mixed methods design to address research question?	Different components of study effectively integrated to answer research question?	Outputs of integration of qualitative/ quantitative components adequately interpreted?	Divergences/inconsistencies between quantitative and qualitative results adequately addressed?	Different components of study adhere to the quality criteria of each tradition of methods involved?
Backman et al. (2018)	N	N	N	Y	N
Drmic et al. (2017)	N	N	N	Y	Y
Edgington et al. (2016)	N	N	N	N	Y

*C* Can’t tell, *N* No, *Y* Yes

### Process Factors Assessed in Quantitative Studies

Among studies that employed quantitative methods (*n* = 22; including randomized and non-randomized, descriptive, and mixed methods designs), the following process factors were identified: family accommodation, parent-therapist alliance, treatment adherence, treatment expectations, treatment readiness, treatment satisfaction, youth involvement, and youth-therapist alliance. Details regarding the measures used to assess each process factor are provided in Table 3. As shown in Table 1, approximately 62% of studies described process factors as indicators of treatment feasibility, without examining process-outcome associations. For example, several studies described homework completion as an indication of treatment adherence (e.g., McNally Keehn et al., 2013; Thomson et al., 2015), but did not report correlations between homework completion and treatment outcome. Eight studies explicitly evaluated the relation between process factors and treatment outcome, and six studies assessed relations among process factors. An overview of study results for each process factor follows below.

**Table 3:** Characteristics of process factor measures used in included studies

Process factor	Measure	Included studies that used measure	Measurement timing	Mode of administration	Scale description
Family accommodation	Family Accommodation Scale (Calvocoressi et al., 1995)	Jassi et al. (2021), Jones and Jassi (2020)	Pre- and post-treatment During treatment	Parent report	13 items assessing family members' accommodation of OCD symptoms Two subscales: Involvement in Compulsions; Avoidance of Triggers 5-point Likert-type scale (0 = Never, 4 = Daily) Overall and subscale sums; Higher scores = greater accommodation Score > 13 indicates clinically significant accommodation
	Pediatric Accommodation Scale (Benito et al., 2015)	Storch et al. (2015)	Pre- and post-treatment	Parent report (clinician-administered)	14 items assessing frequency of family accommodation and impact on child/family functioning Three subscales: Frequency (frequency over previous week, across all items); Parent Impact (4 items); Child Impact (7 items) Three global items: Accommodation from primary and secondary caregivers, and first sibling 4-point Likert-type scale (0 = Never/None; 4 = Always/Extreme) Subscale mean scores; Higher scores = greater accommodation
Parent-therapist alliance	TPOCS-A (McLeod & Weisz, 2005)	Burnham Riosa et al. (2019)	During treatment (early, middle, and late sessions)	Independent-observer-report	9 items assessing two aspects of therapeutic alliance: Therapeutic Bond (6 items) and Task Collaboration (3 items) 6-point Likert-type scale (0 = Not at all; 5 = Great deal) Overall sum; Higher scores = stronger alliance
	TASC-R (Shirk & Saiz, 1992)	Kerns et al. (2018); Klebanoff et al. (2019)	During treatment (following at least two sessions; Klebanoff et al., 2019) Post-treatment (Kerns et al., 2018)	Parent report	12 items assessing two aspects of alliance between therapist and parent: Task (6 items) and Bond (6 items); Kerns et al. (2018) used 7-item version 4-point Likert-type scale (1 = Not at all; 4 = Very much) Overall sum; Higher scores = stronger alliance
	Single-item used in two related studies	Burnham Riosa et al. (2019), Thomson et al. (2015)	During treatment (following each session)	Therapist-report	"How would you describe the quality of the therapeutic relationship during the session with the parent?" 7-point Likert-type scale (1 = Very poor; 7 = Very good) Single-item rating; Higher scores = stronger alliance
Treatment satisfaction	Various unnamed questionnaires used in ten studies (two related)	Backman et al. (2018), Gordon et al. (2015), Hillier et al. (2012), Jassi et al. (2021), Pahnke et al. (2014), Swain et al. (2019), Thomson et al. (2015), Walsh et al. (2018), Weiss et al. (2018), White et al. (2013)	During treatment (following each session; Backman et al., 2018; Thomson et al., 2015; Walsh et al., 2018; Weiss et al., 2018) Post-treatment (Gordon et al., 2015; Hillier et al., 2012; Jassi et al., 2021; Swain et al., 2019; White et al., 2013) NR (Pahnke et al., 2014)	Parent report (Hillier et al., 2012; Jassi et al., 2021; Swain et al., 2019; Thomson et al., 2015; Walsh et al., 2018; Weiss et al., 2018; White et al., 2013) Therapist-report (Thomson et al., 2015; Walsh et al., 2018) Youth-report (Backman et al., 2018; Gordon et al., 2015; Hillier et al., 2012; Jassi et al., 2021; Pahnke et al., 2014; Thomson et al., 2015; Walsh et al., 2018; Weiss et al., 2018; White et al., 2013)	Variable across studies
Youth-therapist alliance	TPOCS-A (McLeod & Weisz, 2005)	Albaum et al. (2020), Brown et al. (2015), Burnham Riosa et al. (2019), Kang et al. (2021)	During treatment (multiple time points per participant for all studies except Brown et al., 2015)	Independent-observer-report	9 items assessing two aspects of therapeutic alliance: Therapeutic Bond (6 items) and Task Collaboration (3 items) 6-point Likert-type scale (0 = Not at all; 5 = Great deal) Overall sum; Higher scores = stronger alliance
	TASC-R (Shirk & Saiz, 1992)	Kang et al. (2021), Kerns et al. (2018), Klebanoff et al. (2019)	During treatment (following at least two sessions; Kang et al., 2021; Klebanoff et al., 2019) Post-treatment (Kerns et al., 2018)	Therapist-report Youth-report	12 items assessing two aspects of alliance between therapist and youth: Task (6 items) and Bond (6 items); Kang et al. (2021) used 13-item version 4-point Likert-type scale (1 = Not at all; 4 = Very much) Overall sum; Higher scores = stronger alliance
	Vanderbilt Therapeutic Alliance Scales Revised, Short Form (Shelef & Diamond, 2008)	Brewe et al. (2021)	During treatment (four time points)	Independent-observer-report	5 items assessing aspects of therapeutic alliance 6-point Likert-type scale (anchors NR) Overall sum; Higher scores = stronger alliance
	Single-item used in two related studies	Burnham Riosa et al. (2019), Thomson et al. (2015)	During treatment (following each session)	Therapist-report	"How would you describe the quality of the therapeutic relationship during the session with the child?" 7-point Likert-type scale—Anchors indicate quality (1 = Very poor; 7 = Very good) Single-item rating; Higher scores = stronger alliance
Youth treatment engagement (i.e., adherence; involvement)	Percentage of homework completion used in four separate studies	Gordon et al. (2015), Lordo et al. (2017), McNally Keehn et al. (2013), White et al. (2013)	During treatment (following each session)	Therapist-report (White et al., 2013) NR (Gordon et al., 2015; Lordo et al., 2017; McNally Keehn et al., 2013)	At least partial completion of between-session assignments (White et al., 2013); Completion not defined (Gordon et al., 2015; Lordo et al., 2017; McNally Keehn et al., 2013) Frequency count; Percentage/number of sessions for which homework was completed
	Rate of participation for between-session skills practice	Pahnke et al. (2014)	During treatment	NR	Number of training occasions at school, between sessions Frequency count
	Single-item rating homework completion used in three related studies	Burnham Riosa et al. (2019); Thomson et al. (2015); Weiss et al. (2018)	During treatment (following each session)	Therapist-report	"Did the client complete the home mission that was assigned?" 3-point scale (1 = None; 2 = Partially; 3 = Fully) Single-item rating; Higher scores = greater adherence
	Single-item rating in-session involvement used in four studies (three related)	Burnham Riosa et al. (2019), Thomson et al. (2015), Weiss et al. (2018), White et al. (2013)	During treatment (following each session)	Therapist-report	Three related studies "How involved was the client during the session?" 5-point Likert-type scale (1 = Completely uninvolved; 5 = Actively involved) Single-item rating; Higher scores = greater involvement White et al. (2013) Item description NR 4-point Likert-type scale (1 = Uninvolved; 4 = Actively involved) Single-item rating; Higher scores = greater involvement
Youth treatment expectations	Treatment Credibility Scale (Borkovec & Nau, 1972)	Backman et al. (2018)	Pre- and post-treatment	Youth-report	5 items assessing expectations of improvement and treatment credibility 11-point visual analogue scale (0 = Low credibility/Not at all; 10 = High credibility/Very much) Overall mean; Higher scores = greater credibility
Youth treatment readiness	Three items used in one study	Albaum et al. (2020)	Pre-treatment	Youth-report	3 items assessing interest, readiness, and willingness to participate in treatment 9-point Likert Scale (0 = Not at all; 8 = Very, very much) Overall mean; Higher scores = greater readiness

*NR* Not reported, *OCD* Obsessive compulsive disorder, *TASC-R* Therapeutic Alliance Scale for Children, Revised, *TPOCS-A* Therapy Process Observational Coding Scheme, Alliance scale

#### Family Accommodation

Family accommodation is described as the tendency for family members to engage in behaviours that aim to prevent the child from experiencing anxiety or aid the child in avoiding anxiety-provoking stimuli (Lebowitz et al., 2012), such as providing unnecessary reassurance or adapting routines to intentionally avoid situations that cause anxiety (Lebowitz et al., 2014). Three studies measured family accommodation of youth anxiety or obsessive–compulsive symptoms (Jassi et al., 2021; Jones & Jassi, 2020; Storch et al., 2015) using two different parent-report measures (details provided in Table 3). All three studies assessed family accommodation pre- and post-treatment, and two studies also assessed family accommodation at multiple timepoints during, and three-months following the end of treatment (Jassi et al., 2021; Jones & Jassi, 2020). All three studies cite evidence for the valid use of the selected measures based on previous research involving youth without autism; however, psychometric properties (e.g., metric of internal consistency) based on the study sample were not reported in any of the studies.

Only one of these studies considered family accommodation in relation to treatment outcome. Storch et al. (2015) assessed parent accommodation of youth anxiety symptoms prior to and following participation in CBT. In comparison to those considered non-responders, families of youth who responded to treatment had a lower frequency of symptom accommodation at the end of treatment, and accommodation had less of an impact on *parents*’ activities and work schedules, family routine, and family distress. There was no significant difference between responders and non-responders in terms of the impact of accommodation on youth treatment outcomes. Family accommodation was the only process factor measured in this study, and thus could not be examined in relation to other process factors. Jassi et al. (2021) reported clinically elevated levels of family accommodation at baseline, which significantly improved by the end of treatment, with changes maintained at 3-month follow-up. However, the authors did not examine the relation between reduction in family accommodation and reduction in OCD symptoms found at post-treatment. Similarly, in their case study of an autistic adolescent receiving modified CBT for treatment-resistant OCD, Jones and Jassi (2020) noted improvements in family accommodation and OCD symptoms, but did not assess the association between the two variables.

#### Parent-Therapist Alliance

Four studies examined therapeutic alliance between parents and therapists; three of these studies assessed parent-therapist alliance at multiple points during treatment (Burnham Riosa et al., 2019; Klebanoff et al., 2019; Thomson et al., 2015), and one measured alliance post-treatment (Kerns et al., 2018). Two studies used the parent-report Therapeutic Alliance Scale for Children, Revised (TASC-R; Shirk & Saiz, 1992) as a measure of alliance, with one reporting adequate internal consistency among scale items (Kerns et al., 2018). Two other studies (derived from the same larger clinical trial; Weiss et al., 2018) relied on therapist-report using a single-item (Burnham Riosa et al., 2019; Thomson et al., 2015). Burnham Riosa et al. (2019) also used the Therapy Process Observational Coding Scheme, Alliance scale (TPOCS-A; McLeod & Weisz, 2005) for independent-observer ratings of parent-therapist alliance, which strongly converged with the single-item therapist ratings. The authors reported good to excellent inter-rater reliability across items, and acceptable to good internal consistencies for TPOCS-A subscales.

Two studies examined therapeutic alliance between parents and therapists, in association with child treatment outcomes. Klebanoff et al. (2019) found parent-therapist alliance, as reported by parents, predicted greater reduction in child anxiety severity post-treatment, with a stronger alliance-outcome correlation for older children (i.e., 10 years or older) compared to younger children (i.e., under 10 years). In contrast, Kerns et al. (2018) did not find a significant relation between parent-therapist alliance and improvements in child anxiety following treatment, nor did they find a difference in parent-therapist alliance between treatment responders and non-responders. In terms of the relation among process factors, Klebanoff et al. (2019) found a moderate correlation between parent-therapist alliance and therapist-reported alliance with youth, whereas Kerns et al. (2018) did not find a significant association. Other studies aimed to examine the psychometric properties of therapeutic alliance measures (Burnham Riosa et al., 2019) or included parent-therapist alliance as an indicator of treatment feasibility (Thomson et al., 2015), and did not report on process-outcome or process-process correlations.

#### Treatment Satisfaction

Treatment satisfaction has not been consistently operationalized, but generally refers to the perceived helpfulness, enjoyment, and/or acceptability of treatment. Treatment satisfaction was the most commonly reported process factor in included studies. Four studies had participants provide ratings of treatment satisfaction following each session, and five studies included a single satisfaction rating provided at the end of treatment. Treatment satisfaction was rated by youth, parents, and/or therapists, with four studies involving a single informant (Backman et al., 2018; Gordon et al., 2015; Pahnke et al., 2014; Swain et al., 2019), and six studies involving multiple informants (Hillier et al., 2012; Jassi et al., 2021; Thomson et al., 2015; Walsh et al., 2018; Weiss et al., 2018; White et al., 2013). Measures were typically developed to assess satisfaction for the specific

intervention program being delivered, and thus tended to vary across studies. None of the studies reported on psychometric properties of treatment satisfaction measures.

The majority of studies that reported on treatment satisfaction did not describe process-outcome or process-process associations, but reported on satisfaction as an indication of treatment feasibility. In general, participants reported being satisfied with the treatment they received. Only two studies measured the relation between treatment satisfaction and mental health outcomes. Walsh et al. (2018) examined the association between youth and parent ratings of treatment acceptability, and improvements in anxiety following participation in CBT. The authors found that for sessions focused on exposure to anxiety-provoking stimuli, acceptability ratings from both parent and youth respondents negatively predicted anxiety severity following treatment. For sessions that focused on psychoeducation, neither parent nor youth ratings of acceptability predicted treatment outcome. Across all sessions, parents indicated greater satisfaction with treatment compared to youth. Swain et al. (2019) also assessed satisfaction in treatment targeting emotional problems and emotion regulation, and found that treatment satisfaction did not differ between treatment responders and non-responders.

#### Youth-Therapist Alliance

Eight studies measured therapeutic alliance between therapists and youth; six of which measured alliance at multiple time points during treatment, one which measured at one point during treatment (Brown et al., 2015), and one which measured post-treatment (Kerns et al., 2018). Four studies used independent-observer ratings based on the TPOCS-A (McLeod & Weisz, 2005), which collectively reported good to excellent inter-rater reliability across items (Albaum et al., 2020; Brown et al., 2015; Burnham Riosa et al., 2019; Kang et al., 2021), good to excellent internal consistencies across subscales (Albaum et al., 2020; Burnham Riosa et al., 2019), temporal stability of ratings (Kang et al., 2021), and strong convergence with single-item therapist ratings of alliance (Burnham Riosa et al., 2019), although no convergence with youth-rated alliance (Kang et al., 2021). Brewe et al. (2021) also relied on independent-observer reports of youth-therapist alliance based on the Vanderbilt Therapeutic Alliance Scales Revised (Shelef & Diamond, 2008), which was found to have good internal consistency and excellent interrater reliability. Three studies used therapist and/or youth reports of alliance based on the TASC-R (Shirk & Saiz, 1992), and reported good to excellent internal consistencies (Kang et al., 2021; Kerns et al., 2018; Klebanoff et al., 2019), temporal stability (Kang et al., 2021), and moderate convergence between therapist and youth ratings of alliance (Kerns et al., 2018; Klebanoff et al., 2019). The two studies derived from the same larger clinical trial also used a single-item to assess therapist ratings of youth-therapist alliance as part of a psychometric evaluation of therapeutic alliance measures (Burnham Riosa et al., 2019), or as an indicator of treatment feasibility (Thomson et al., 2015).

Five studies focused on the relation between youth-therapist alliance and treatment outcome (Albaum et al., 2020; Brewe et al., 2021; Kang et al., 2021; Kerns et al., 2018; Klebanoff et al., 2019). Across studies, there was some association between youth-therapist alliance and improvement in mental health outcomes for autistic youth following psychosocial intervention. Two studies employed measures that relied on therapist and youth reports of therapeutic alliance (Kerns et al., 2018; Klebanoff et al., 2019), which found that higher therapist ratings of youth-therapist alliance were related to greater reduction in parent- and clinician-rated youth anxiety and global symptom severity. Comparatively, results from both studies indicated that youth-reported alliance was not significantly related to treatment outcome. Kerns et al. (2018) also found that therapist-reported alliance was stronger for treatment responders compared to non-responders (categorized based on clinician-ratings of symptom improvement), but there was no significant difference between groups in terms of youth-reported alliance. These findings were consistent with results from Kang et al. (2021), who reported a non-significant association between youth-reported alliance and changes in youth self-reported social anxiety. Three studies (Albaum et al., 2020; Brewe et al., 2021; Kang et al., 2021) used measures of therapeutic alliance that relied on independent-observer ratings and found some associations with improvements in emotion regulation. Brewe et al. (2021) indicated that stronger observer-reported alliance was associated with reduced feelings of dysphoria, but not with changes in emotional reactivity, while Albaum et al. (2020) found that observer ratings of therapeutic alliance taken late in treatment, but not early, were related to improvements in parent-reported emotional lability and negativity post-treatment. However, observer-reported alliance did not predict youth-reported treatment outcomes (Albaum et al., 2020; Kang et al., 2021). Two studies further examined specific components of therapeutic alliance relative to treatment outcomes with mixed results; findings from Albaum et al. (2020) indicated task-collaboration, but not therapeutic bond, was a significant predictor of treatment outcome, while Klebanoff et al. (2019) found that bond predicted treatment outcome, but agreement on therapeutic tasks did not. Notably, the two studies differed in terms of reporting source (i.e., therapist-report vs. independent-observer) and the mental health outcome assessed (i.e., anxiety and global symptom severity vs. emotion dysregulation). Two other studies reported on associations between youth-therapist alliance and other process factors. Observer-reported alliance was not related to pre-treatment youth ratings of treatment readiness (Albaum et al., 2020). Burnham Riosa et al. (2019) found moderate to strong correlations between observer ratings of alliance and therapist-rated treatment adherence (i.e., homework completion) and youth involvement in therapy sessions.

#### Youth Treatment Engagement

Eight studies assessed different aspects of treatment engagement, none of which examined associations between engagement and outcome. All eight studies described indicators of treatment adherence, including rates of homework completion or between-session skills practice, and collected information regarding adherence following each therapy session. Adherence was most often rated using therapist-report on a single-item with “yes/no” or similar categorical response format (e.g., “Did the client complete the assigned homework?”). Four studies also included therapist-reported youth in-session involvement; three of which were derived from the same sample (Burnham Riosa et al., 2019; Thomson et al., 2015; Weiss et al., 2018). As with treatment adherence, in-session involvement was measured using a single-item (e.g., “How involved was the client during the session?”) completed by therapists at the end of each session. None of the included studies reported analytic results of treatment engagement beyond basic descriptive statistics (i.e., frequency; mean and SD).

#### Youth Treatment Expectations

Treatment expectation was assessed in one study (Backman et al., 2018), which measured adolescents’ perceptions of treatment credibility regarding the autism-specific psychoeducation program being provided. The authors did not examine the relation between treatment credibility and mental health outcome but found a large- sized improvement in treatment credibility from pre- to post-intervention (*η*^*2*^ = 0.30). Psychometric information about the measure used to assess treatment credibility was not provided.

#### Youth Treatment Readiness

One study included a measure of youth treatment readiness (Albaum et al., 2020), which was assessed pre-treatment using three items that asked children to rate their interest, readiness, and willingness to participate in therapy. The authors examined the association between treatment readiness and youth-therapist alliance both early (i.e., first half) and late (i.e., second half) in treatment, and found no significant relation. Psychometric information of validity and reliability were not provided, and the authors did not evaluate treatment readiness in relation to outcome.

### Process Factors Described in Qualitative Studies

Four of the included studies conducted qualitative analyses to evaluate participants’ experiences with mental health interventions (Chlebowski et al., 2018; Drmic et al., 2017; Edgington et al., 2016; London et al., 2020), which resulted in several emerging themes of process-related factors. Three studies identified themes related to parent involvement in mental health treatment for autistic youth. For example, strong, consistent support from stakeholders (including parents), such as parent enthusiasm for their child’s treatment, was described as an important factor for facilitating and implementing CBT for anxiety in autistic adolescents (Drmic et al., 2020). Themes related to challenges with parent involvement also emerged, such as difficulties with obtaining information from adolescents when parents are not directly involved in treatment (Edgington et al., 2016), and different perceptions between therapists and parents in terms of the expected role parents play in mental health treatment for children (e.g., expecting child to take part in sessions independently, without parent involvement; Chlebowski et al., 2018). Youth motivation was a relevant theme in considering the feasibility of implementing an intervention program, and participants’ acceptability and skill application. Identifying motivated adolescents to participate in treatment was important for establishing positive rapport and facilitating enjoyable sessions (Drmic et al., 2020), and self-motivation was relevant for unprompted use of coping strategies (Edgington et al., 2016). Relatedly, a lack of readiness from adolescents to participate in groups was described as a barrier for coaches to facilitate sessions smoothly (Drmic et al., 2020). Other process factors that were mentioned in qualitative themes included group cohesion (e.g., importance of group dynamics; Edgington et al., 2016), therapist direct influence skills (e.g., ability to carry out program delivery; Drmic et al., 2020), the relationship between parents and therapists (Chlebowski et al., 2018), and youth engagement in relation to treatment progress (London et al., 2020).

## Discussion

Therapeutic process factors are known predictors of treatment outcome and may serve as mechanisms for fostering change in psychosocial interventions. Research evaluating process factors has largely involved youth without autism, restricting the generalizability of findings to interventions that target mental health concerns for autistic children and adolescents. The current literature search yielded 25 studies that involved autistic youth (i.e., under 18 years of age) who took part in psychosocial interventions to address the emotional and psychological aspects of mental health issues, such as anxiety, depression, or related challenges (e.g., emotion dysregulation, stress-related behaviour). Across studies, various factors were described or assessed that can be classified into three overarching domains: relational factors (e.g., therapeutic alliance); expectations, readiness, and satisfaction; and treatment engagement (e.g., involvement; adherence).

### Relational Factors

Relational factors comprise constructs that describe different aspects of the relationships between therapists, clients, and caregivers, both within and outside therapy sessions. Consistent with the non-autism literature (Fjermestad et al., 2009; Karver et al., 2006), relational factors appear to be the most well-understood aspect of the therapeutic process in mental health treatment for autistic youth. Several studies examined therapeutic alliance between therapists and autistic youth. Therapeutic alliance refers to the working relationship between therapist and client, based on bond, collaboration on therapeutic tasks, and agreed upon treatment goals (Bordin, 1979). Preliminary psychometric evidence suggests that youth-therapist alliance can be validly assessed through different informants (e.g., youth, therapist, independent-observer) at multiple points during treatment, making it possible to conduct longitudinal analyses using multiple perspectives. Within the general child literature, variation in alliance-outcome associations has been attributed in part to reporting source (e.g., youth vs. therapist; Karver et al., 2018). Researchers should, therefore, consider incorporating measures that rely on multiple perspectives when evaluating alliance with autistic clients to help determine the predictive validity of ratings provided by different informants. Findings across studies indicate that it is not only possible for clinicians to form a strong working relationship, but that therapeutic alliance may be an important contributor to improvements in anxiety (Kerns et al., 2018; Klebanoff et al., 2019) and emotion regulation (Albaum et al., 2020; Brewe et al., 2021) for autistic children and adolescents. Therapeutic alliance with autistic youth was also found to be positively associated with youth involvement during therapy sessions, and treatment adherence outside of sessions (Burnham Riosa et al., 2019), though further research is needed to understand the transactional pattern that occurs between these factors over the course of therapy. Given the inherent social-communication challenges associated with autism that can make it difficult for youth to form meaningful relationships, it is critical to consider therapist factors that may contribute to establishing and maintaining alliance with these clients. For example, clinicians’ lack of knowledge and experience, poor competence, and low confidence about working with autistic adults have been identified as barriers to treatment for these clients (Maddox et al., 2020). A recent study also found that therapists are less likely to treat autistic youth compared to youth with ADHD, which was partly explained by differences in therapist attitudes about working with each population, their knowledge about mental health for autistic clients, and the normative pressures they felt about treating autistic youth (Roudbarani et al., in press). In light of the association between therapist attitudes and intention to treat, and the proposed theoretical connection between therapist attitudes and process factors (Karver et al., 2005), it is important to evaluate how these attitudes may influence relational aspects of the therapeutic process that are relevant for treatment outcome. For instance, researchers may aim to answer questions such as: *Do more favourable therapist attitudes predict stronger therapeutic alliance? Does the formation of therapeutic alliance strengthen therapist attitudes about working with autistic clients? Are more favourable therapist attitudes related to greater improvement in treatment outcome, and is this association mediated by the quality of therapeutic alliance?* Addressing these research questions may involve assessing therapist attitudes prior to beginning therapy, and then repeatedly measuring attitudes and therapeutic alliance over the course of treatment to determine whether there is a shift in therapist attitudes, and if there is bidirectional link between attitudes and alliance. In addition, therapists may find it beneficial to have youth and parents complete brief measures of therapeutic alliance (e.g., TASC-R) to monitor their perspectives on the relationship, and then actively work to strengthen the relationship with autistic clients by addressing areas of concern that have been indicated.

Review findings suggest that family accommodation may also be a relevant relational factor to consider when addressing mental health concerns with this population. Family accommodation refers to parent and other family members’ behaviour that abets the child in avoiding anxiety-inducing experiences (Lebowitz et al., 2012, 2014). Previous reviews of the general child literature (e.g., Karver et al., 2006) have not considered family accommodation as part of the therapy process, though it may be a pertinent factor to consider when parents and family members are involved in a child’s treatment. For youth without autism, family accommodation is positively related to child anxiety levels, and reductions in family accommodation have been linked to greater symptom improvement (Lebowitz et al., 2014; Merlo et al., 2009). Three studies identified through the review indicated progressive reduction in parent-reported family accommodation over the course of therapy (Jassi et al., 2021; Jones & Jassi, 2020; Storch et al., 2015), with one study finding a negative association between family accommodation and treatment response (i.e., treatment responders reported lower levels of accommodation compared to non-responders; Storch et al., 2015). Shifts in family accommodation may be particularly relevant to the therapeutic process when parents are involved in their child’s treatment, as is often the case for autistic youth (Reaven, 2011). However, based on literature currently available, it is still unclear whether *changes* in family accommodation are related to changes in mental health outcomes, and if family accommodation is associated with other aspects of the therapeutic process, such as parent-therapist alliance or parent beliefs about treatment.

### Expectations, Readiness and Satisfaction

Client expectations regarding the efficacy, relevance, and importance of treatment, as well as their readiness or motivation to participate, are thought to be key factors related to treatment attendance and adherence (Karver et al., 2005; King et al., 2014). Although treatment expectations have been considered pre-treatment characteristics (Karver et al., 2005), evidence from the current review suggests that beliefs about treatment may not necessarily be fixed, as Backman et al. (2018) found that participants’ perceptions of treatment credibility shifted over the course of therapy. Within the context of interventions for children and adolescents without autism, both youth *and* parent expectations and readiness may have a dynamic association with treatment participation, in turn influencing therapeutic outcome (Karver et al., 2006). Minimal research has explored these processes for autistic youth, either in terms of construct validity or treatment outcome. None of the studies identified in the review assessed parent expectations and willingness to participate in their child’s treatment. Qualitative analyses indicate that youth motivation may be relevant to therapist capacity to establish rapport and facilitate sessions, and treatment adherence on the part of the youth outside of therapy sessions. Parent expectations regarding their role in their child’s treatment was also described as pertinent to the provision of mental health treatment for autistic adolescents (Chlebowski et al., 2018). As noted above, Backman et al. (2018) quantitatively assessed youth perceptions of treatment credibility during the receipt of psychoeducation about autism, and found that perceived credibility improved over the course of the intervention. However, the authors did not examine whether credibility was associated with changes in youth anxiety or depression. Similarly, a second study assessed children’s readiness and willingness to take part in treatment prior to participating in CBT and found a non-significant relation between treatment readiness and child-therapist alliance (Albaum et al., 2020). The authors reported a considerable range in the degree of child readiness across participants, suggesting that autistic youth who take part in mental health intervention likely vary in their willingness and commitment to participate; however, they did not assess the association between treatment readiness and changes in mental health outcomes following treatment completion. Thus, there is a lack of empirical evidence available on the expectations autistic youth and their parents have regarding therapy, and how motivation or readiness for treatment is related to the therapy process and positive outcomes. It may be worthwhile for researchers to evaluate client beliefs about therapy prior to starting treatment, and track expectations and motivation during treatment. Findings from research of this nature could help to determine whether there are common patterns in the ways autistic clients’ beliefs about therapy change over the course of treatment, and if certain trajectories are associated with better outcomes. Establishing an evidence base on the connection between clients’ beliefs and treatment outcome could help inform adaptations to therapeutic techniques, such as motivational interviewing (Feinberg et al., 2021; Rogers et al., 2019), which can be used by clinicians to promote expectations, motivation, and treatment readiness for autistic youth and their parents.

Related to client expectations is the extent to which these expectations are met, and whether they are satisfied in their therapeutic experience, based on perceived helpfulness, relevance, and enjoyment. It has been hypothesized that treatment satisfaction may contribute to active involvement and better compliance, though the mechanistic pathway between these process-related variables is poorly understood (Karver et al., 2005). Within the autism literature, it appears that treatment satisfaction is often reported as an indicator of overall feasibility or acceptability of the intervention. Satisfaction was often analyzed descriptively, with various studies reporting high ratings of satisfaction from both parents and youth. Only two studies reported on relations between treatment satisfaction and symptom improvement (Swain et al., 2019; Walsh et al., 2018), with mixed results. Researchers who are evaluating interventions and choose to include measures of treatment satisfaction should consider exploring shifts in satisfaction that may occur throughout treatment, and possible interactions with other key therapeutic processes (e.g., compliance, adherence) that may contribute to variation in treatment effectiveness.

### Treatment Engagement

Engagement in treatment is considered critical for successful outcomes (Becker et al., 2018). Within the general youth literature, the term “engagement” tends to be used interchangeably with “involvement” or “participation”, and refers to the active, effortful, and collaborative role that youth and parents have during and between therapy sessions (Karver et al., 2005). Research has yet to explore treatment engagement in relation to outcome within the context of mental health intervention for autistic youth. Several studies identified through the review provided quantitative descriptions of treatment adherence, generally operationalized as homework completion outside of sessions (e.g., Gordon et al., 2015; Pahnke et al., 2014); however, homework completion or between-session practice was typically dichotomized as “complete” or “incomplete”, failing to consider other factors, such as ease or difficulty of assigned practice, that may be relevant to the overarching construct of engagement. Youth involvement within therapy sessions was also described in several studies (e.g., Weiss et al., 2018; White et al., 2013), but relied solely on global therapist ratings on a single-item. Developing valid and reliable measures that consider nuanced behavioural indications of in-session participation can lead to greater understanding of how engagement contributes to treatment success, and can equip clinicians with the knowledge and skills needed to promote participation during sessions with autistic clients.

Parent involvement was an overarching theme in multiple studies that employed qualitative methods (Chlebowski et al., 2018; Drmic et al., 2017; Edgington et al., 2016). Direct parent involvement may be particularly important for parents to learn how to best assist their child in practicing the skills being taught (Drmic et al., 2017; Edgington et al., 2016). For younger children, parents may be expected to take on various roles in their children’s therapy, such as co-therapists. Incongruence between therapist and parent expectations about the extent of parent involvement can potentially hinder the quality of parent participation both within and outside of sessions (Chlebowski et al., 2018). Prior to beginning therapy, establishing clear, agreed upon expectations between therapists and parents about participation may augment engagement in their child’s treatment. Some studies have empirically examined therapeutic alliance between therapists and caregivers of autistic youth who participate in their children’s therapy. There is emerging support for the valid measurement of parent-therapist alliance; however, findings are inconsistent in terms of process-outcome associations. Klebanoff et al. (2019) found parent-therapist alliance was associated with greater reduction in anxiety post-treatment, whereas Kerns et al. (2018) did not find a significant process-outcome association. Given that parent involvement is a recommended modification to psychosocial intervention for autistic youth (Reaven, 2011; Walters et al., 2016), it is important to establish stronger empirical knowledge about how parents contribute to their child’s treatment success.

### Limitations

Review findings should be interpreted within the context of the study’s limitations. The current review only offers a narrative synthesis of process factors; meta-analysis of effect sizes was not calculated, thus preventing comparison of potential process-outcome associations. This review only included studies published in English, and additional empirical evidence regarding process factors may be available in other languages. In regard to client characteristics, several studies included samples for which a portion of participants were over the age of 18 years (Backman et al., 2018; Brewe et al., 2021; Hillier et al., 2012; London et al., 2020; Pahnke et al., 2014), but did not conduct analyses to evaluate potential effects of age (e.g., separating < 18 vs. 18 +; including age as covariate). Samples were also limited in terms of ethnic diversity, with most studies reporting that the majority of the sample comprised participants who identified as White/Caucasian. Finally, results are based on samples that almost exclusively involve autistic youth without co-occurring intellectual disabilities and may not be equally relevant for mental health treatment for youth with limited cognitive abilities and adaptive skills.

## Conclusions

There is a limited, albeit growing, body of high-quality research evaluating the role of process factors in the treatment of mental health issues for young autistic people. Researchers have begun to examine constructs related to therapeutic relationships, treatment expectations and satisfaction, and youth engagement throughout treatment, though there is still little understanding of how exactly these factors contribute to therapeutic outcomes. Future studies should continue to focus on better understood process factors, such as therapeutic alliance, and address gaps around less well-known factors, such as in-session participation and parent involvement. Specifically, researchers should examine process-outcome associations, providing metrics of effect size that can eventually be included in meta-analyses once a sufficient pool of results exists. Considering associations amongst process factors over the course of therapy, such as the youth-therapist and parent-therapist alliance, or youth and parent engagement, may also be a fruitful research direction to pursue. Further, validation of measurement tools that accurately capture process-related constructs within the context of treatment for autistic youth is necessary. It will be important for researchers to explore process factors with samples of autistic youth who represent the full spectrum of functioning in regard to social-communicative skills, and intellectual and adaptive functioning. Immediate and direct implications for clinical practice include establishing a strong therapeutic alliance with autistic youth and their parents, clarifying expectations about parent involvement in their child’s therapy, and encouraging active involvement from both youth and parents during and between therapy sessions. Greater understanding of therapy processes can provide a knowledge base that allows for evidence-informed strategies to be implemented by clinicians to promote positive expectations, relationships, and engagement. By addressing process-related barriers, therapists may be able to improve the effectiveness of mental health treatment for autistic youth.

## Supplementary Information

Below is the link to the electronic supplementary material.

Supplementary file1 (PDF 54 kb)

## Data Availability

Data sharing is not applicable to this article as no new data were created and no datasets were generated or analysed during the current study. Additional information is available from the corresponding author upon request.

## Footnotes

1 The term “youth” is used here to describe young people under the age of 18 years. “Children” refers to youth 12 years of age and younger, and “adolescents” refers to youth between 13 and 17 years of age.
