# Supplementary material for: A Systematic Review of Therapeutic Process Factors in Mental Health Treatment for Autistic Youth
Source: Clin Child Fam Psychol Rev. 2022 Aug 24;26(1):212–41. doi: 10.1007/s10567-022-00409-0 (PMC9879813; doi:10.1007/s10567-022-00409-0)
Supplement: Supplementary file 1 — Supplementary file1 (PDF 54 kb) [file 10567_2022_409_MOESM1_ESM.pdf]

Supplemental Table 1. Search strategy	
Database	Search strategy
MEDLINE	1. Autistic Disorder/<br>2. Autism Spectrum Disorder/<br>3. Asperger Syndrome/<br>4. 1 or 2 or 3<br>5. Child/<br>6. Adolescent/<br>7. Pediatrics/<br>8. 5 or 6 or 7<br>9. Therapeutic Alliance/<br>10. Empathy/<br>11. goals/<br>12. collaboration.mp.<br>13. resistance.mp.<br>14. therap* relationship.mp.<br>15. positive regard.mp.<br>16. congruence.mp.<br>17. therap* rupture.mp.<br>18. impasses.mp.<br>19. therapy repair.mp.<br>20. Self Disclosure/<br>21. Countertransference/<br>22. relational interpretation.mp.<br>23. Motivation/<br>24. Patient Preference/<br>25. Assimilation.mp.<br>26. attachment.mp.<br>27. engagement.mp.<br>28. treatment induction.mp.<br>29. openness.mp.<br>30. bond.mp.<br>31. comfort.mp.<br>32. Cooperative Behavior/<br>33. treatment difficulty.mp.<br>34. treatment involvement.mp.<br>35. willingness.mp.<br>36. Patient Participation/<br>37. treatment transaction.mp.<br>38. warmth.mp.<br>39. Trust/<br>40. therap* process.mp.<br>41. 9 or 10 or 11 or 12 or 13 or 14 or 15 or 16 or 17 or 18 or 19 or 20 or 21 or 22 or 23 or 24 or 25 or 26 or 27 or 28 or 29 or 30 or 31 or 32 or 33 or 34 or 35 or 36 or 37 or 38 or 39 or 40<br>42. 4 and 8 and 41

PsycINFO	(child* OR mainsubject(Pediatrics) OR noft(youth) OR noft(adolescen*) OR noft(kid)) AND mainsubject(Autism Spectrum Disorders) AND ((mainsubject(Therapeutic Alliance) OR mainsubject(Empathy) OR mainsubject(Goals) OR mainsubject(Collaboration) OR mainsubject(Resistance) OR mainsubject(Psychotherapeutic Resistance) OR mainsubject(Self-Disclosure) OR mainsubject(Countertransference) OR mainsubject(Expectations) OR mainsubject(Preferences)) OR (mainsubject(Assimilation (Cognitive Process)) OR mainsubject(Attachment Behavior) OR mainsubject(Openness to Experience) OR mainsubject(Bonding (Emotional)) OR mainsubject(Cooperation) OR mainsubject(Involvement) OR mainsubject(Participation) OR mainsubject(Trust) OR mainsubject(Therapeutic Processes) OR mainsubject(Treatment Process AND Outcome Measures)) OR (noft(therap* relationship) OR noft(positive regard) OR noft(congruence) OR noft(rupture) OR noft(impasses) OR noft(repair) OR noft(relational interpretation) OR noft(treatment induction) OR noft(comfort) OR noft(treatment difficulty)) OR (noft(willingness) OR noft(treatment transaction) OR noft(warmth)))
PubMed	(((autism spectrum disorder[MeSH Terms]) OR (autistic disorder[MeSH Terms]) OR (asperger syndrome[MeSH Terms])) AND (("child"[MeSH Terms]) OR (pediatrics[MeSH Terms]) OR (adolescent[MeSH Terms]))) AND ((("therapeutic alliance"[MeSH Terms]) OR (empathy[MeSH Terms]) OR (goals[MeSH Terms]) OR ("self disclosure"[MeSH Terms]) OR (countertransference[MeSH Terms]) OR (motivation[MeSH Terms]) OR ("patient preference"[MeSH Terms]) OR ("object attachment"[MeSH Terms]) OR ("patient comfort"[MeSH Terms]) OR ("patient compliance"[MeSH Terms]) OR ("patient participation"[MeSH Terms]) OR (trust[MeSH Terms]) OR ("psychotherapeutic processes"[MeSH Terms]) OR (process assessment health care[MeSH Terms]) OR (collaboration[Title/Abstract]) OR (resistance[Title/Abstract]) OR ("therapy relationship"[Title/Abstract]) OR ("positive regard"[Title/Abstract]) OR (congruence[Title/Abstract]) OR ("rupture"[Title/Abstract]) OR (impasses[Title/Abstract]) OR ("repair"[Title/Abstract]) OR ("relational interpretation"[Title/Abstract]) OR (assimilation[Title/Abstract]) OR (engagement[Title/Abstract]) OR ("treatment induction"[Title/Abstract]) OR (openness[Title/Abstract]) OR (bond[Title/Abstract]) OR ("treatment difficulty"[Title/Abstract]) OR ("treatment involvement"[Title/Abstract]) OR (willingness[Title/Abstract]) OR (warmth[Title/Abstract])))
